# Antiviral and Antibacterial Sulfated Polysaccharide–Chitosan Nanocomposite Particles as a Drug Carrier

**DOI:** 10.3390/molecules28052105

**Published:** 2023-02-23

**Authors:** Ai-Yi Yin, Junpeng Xu, Chii-Shen Yang, Shan-hui Hsu

**Affiliations:** 1 Institute of Polymer Science and Engineering, National Taiwan University, No. 1, Sec. 4 Roosevelt Road, Taipei 10617, Taiwan; 2 Department of Biochemical Science and Technology, National Taiwan University, No. 1, Sec. 4 Roosevelt Road, Taipei 10617, Taiwan; 3 Institute of Cellular and System Medicine, National Health Research Institutes, No. 35 Keyan Road, Miaoli 35053, Taiwan

**Keywords:** *Arthrospira*, sulfated polysaccharide, nanoparticles, antibacterial, antiviral, drug delivery

## Abstract

Drug delivery system (DDS) refers to the method of delivering drugs to the targeted sites with minimal risk. One popular strategy of DDS is using nanoparticles as a drug carrier, which are made from biocompatible and degradable polymers. Here, nanoparticles composed of *Arthrospira*-derived sulfated polysaccharide (AP) and chitosan were developed and expected to possess the capabilities of antiviral, antibacterial, and pH-sensitive properties. The composite nanoparticles, abbreviated as APC, were optimized for stability of morphology and size (~160 nm) in the physiological environment (pH = 7.4). Potent antibacterial (over 2 μg/mL) and antiviral (over 6.596 μg/mL) properties were verified in vitro. The pH-sensitive release behavior and release kinetics of drug-loaded APC nanoparticles were examined for various categories of drugs, including hydrophilic, hydrophobic, and protein drugs, under different pH values of the surroundings. Effects of APC nanoparticles were also evaluated in lung cancer cells and neural stem cells. The use of APC nanoparticles as a drug delivery system maintained the bioactivity of the drug to inhibit the proliferation of lung cancer cells (with ~40% reduction) and to relieve the growth inhibitory effect on neural stem cells. These findings indicate that the pH-sensitive and biocompatible composite nanoparticles of sulfated polysaccharide and chitosan well keep the antiviral and antibacterial properties and may serve as a promising multifunctional drug carrier for further biomedical applications.

## 1. Introduction

Drug delivery system (DDS) refers to the delivery of drug compounds to the target site and achieving the desired therapeutic effect in a safe and minimal-risk manner [1]. The important goal of DDS is to increase the efficiency of drug use, improve the effectiveness of treatment, and reduce the side effects caused by excessive drug toxicity. Such systems are designed through drug molecules, manufacturing drug-loaded particles, and the optimization of storage systems [2]. Various organic/inorganic nanoparticles employed as drug carriers have shown great potential to improve the therapeutic efficacy and reduce adverse side effects [3,4]. In comparison to non-biodegradable materials, a biocompatible and degradable polymer can be excreted, which avoids the potential risk of toxicity. Polysaccharides are a group of structurally diverse biopolymers that are mostly extracted from microorganisms or plants and can have various biological properties [5]. Nearly all polysaccharides possess biocompatibility and degradability. Therefore, many polysaccharides were selected for development as drug carriers of different forms, such as in the form of nanoparticles [6,7]

Sulfated polysaccharide (SP) is one of the diverse branches of polysaccharides. SP refers to a family of natural and semisynthetic acidic polysaccharides, which are formed by the substitution of hydroxyl groups on monosaccharides in macromolecular chains by sulfate groups [8]. SP owns cytocompatibility because of the structural similarity to mucopolysaccharides of the human body [9,10,11]. In particular, SP exhibits an exclusive structure that exerts antiviral effects. The interaction of polysaccharides with sulfate groups blocks the viral life cycles before infection or by inhibiting its replication [12]. Because of the special biological properties, SP has strong potential for development as an antiviral drug [13]. Nevertheless, the medicinal application of SP is mostly as a drug. Some SP-based polymer networks have been reported very recently, but they still need to be complemented by additional conditions, such as the presence of an originally gellable matrix [14,15] or metal ionization [16]. This limitation is probably imposed by the structure of the SP [17]. A recent study verified the possibility of *Arthrospira*-derived SP (AP) as a part of the drug-loading nanoparticles [18]. The high-purity AP is featured with high molecular weight (~300 kDa) and good water solubility. Owing to negatively charged sulfate groups, AP can interact with the cationic polymer such as chitosan (CS) and self-assemble in the nanoparticulate form. However, the detailed mechanism and an optimized process for drug loading/release are yet to be established.

CS, as a biocompatible and biodegradable cationic polymer with an additional antibacterial function, has served as an adjuvant in drug delivery [19]. CS is a linear polysaccharide composed of glucosamine (deacetylated units) and N-acetylglucosamine (acetylated units) randomly distributed and combined through glycosidic bonds [20]. It has been extensively studied as biomedical materials [21,22]. The application of CS is limited due to its insolubility in water, and CS alone as a drug carrier may have shortcomings, such as insufficient drug loading and uncontrollable drug release [23]. CS combined with other molecules or polymers can be an effective method to prepare drug carriers. For example, CS and heparin (a mucopolysaccharide) formed complex nanoparticles to improve drug absorption [24,25].

AP is negatively charged, which makes it possible to interact with CS through electrostatic force to form composite nanoparticles. Moreover, the water solubility of AP may expand the applications of CS. In this work, we developed pH-sensitive composite nanoparticles using AP and CS. The combination of these two polymers was expected to have antiviral and antibacterial effects. The size and zeta potential of the composite nanoparticles were optimized through the preparation process. The thermal properties of the nanoparticles and the behavior of the nanoparticles under different pH were also investigated. The nanoparticles were then encapsulated with hydrophilic and hydrophobic model drugs as well as a mass-produced photosensitive protein [26] as a protein drug to examine the possible encapsulation and release profiles of various types of drugs. Moreover, the potential functionality of the composite nanoparticles in modulating cell proliferation was evaluated.

## 2. Results

### 2.1. Properties of Composite Nanoparticles and Optimization

**Figure 1:**
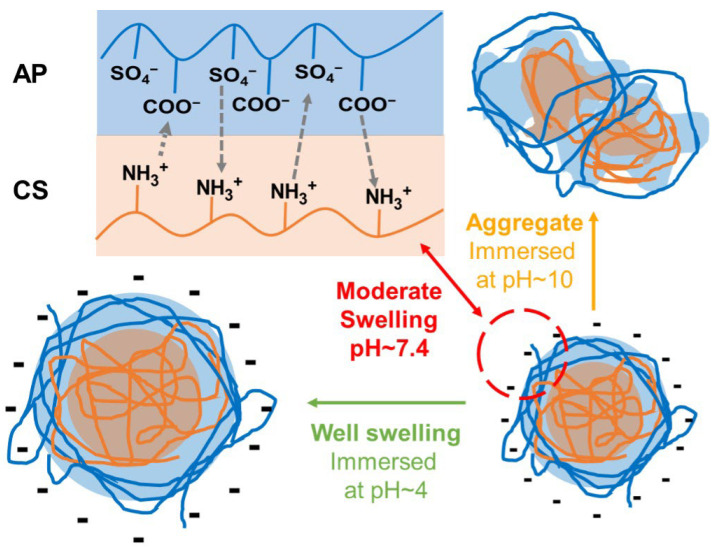
A schematic diagram for the formation of the APC nanoparticles and the potential structural changes in response to the surroundings of different pH values.

The composite nanoparticles, abbreviated as APC nanoparticles, were prepared at room temperature by mixing AP solution and CS solution through optimized procedures. The mechanism for the formation of the APC nanoparticles and the potential structural changes in response to surroundings with different pH values are shown in Figure 1. The abbreviated names and compositions of various homogeneous APC nanoparticles are summarized in Table 1. A further upsurge of the CS to 1.5 mg/mL in preparation of the APC was also evaluated (Figure S1), but the nanoparticles generated were not stable and immediately aggregated after mixing to form large, inhomogeneous precipitates.

**Figure 2:**
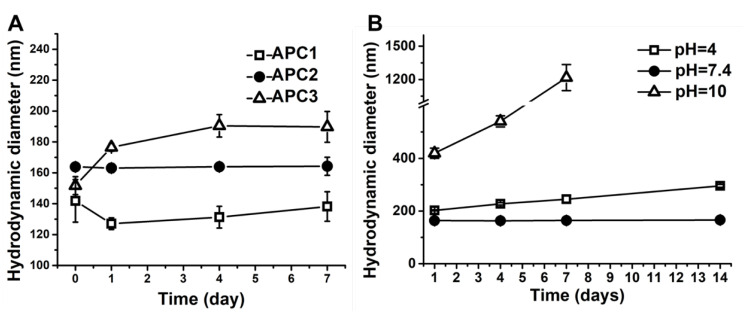
The stability and pH sensitivity of APC nanoparticles as detected by the hydrodynamic diameters versus immersion time. (**A**) The changes of hydrodynamic diameters were detected against the immersion time in APC nanoparticles with different compositions in a period of 7 days at 4 °C. (**B**) The changes of hydrodynamic diameters were detected for APC2 nanoparticles under different pH values in a period of 14 days at 25 °C.

**Figure 3:**
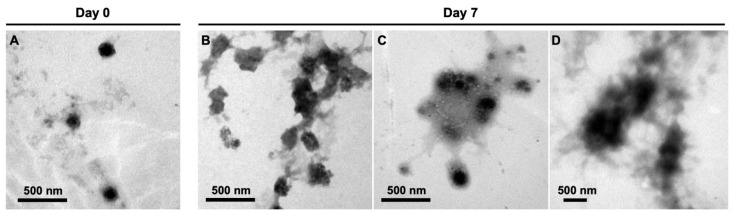
TEM images of APC nanoparticles. APC2 nanoparticles were observed initially at day 0 (**A**) and were immersed for 7 days at pH 4 (**B**), pH 7 (**C**), and pH 10 (**D**) for observation.

The successfully prepared APC nanoparticles were re-suspended in deionized (DI) water for characterization. The changes of hydrodynamic diameters against the time of immersion in DI water of APC nanoparticles are shown in Figure 2A. The initial zeta potentials and polydispersity index (PDI) of various APC nanoparticles are summarized in Table 2. At day 0, the hydrodynamic diameters of APC1 and APC3 nanoparticles were smaller than that of APC2 nanoparticles. After 7 days, APC2 nanoparticles had the most stable hydrodynamic diameter (~164 nm) versus time compared to the other two groups. The hydrodynamic diameter of APC3 nanoparticles gradually increased to 190 nm. The hydrodynamic diameter of APC1 nanoparticles slightly decreased on the first day and then increased in the following days, yet overall the size of APC1 (~138 nm) was still smaller than that of APC2 nanoparticles. In addition, the variation of hydrodynamic diameter for APC2 nanoparticles under surroundings of different pH values is presented in Figure 2B. After 14 days, the diameter of APC2 under neutral conditions (pH = 7.4) remained nearly unchanged at about 166 nm. Under the acidic (pH = 4.0) condition, the hydrodynamic diameter of APC2 nanoparticles increased slowly and reached about 295 nm after 14 days. Under the alkaline (pH = 10.0) condition, the hydrodynamic diameter of APC2 nanoparticles became four times larger than that in the neutral solution (pH = 7.4). A large amount of non-suspended precipitates from APC2 in alkaline surrounding (pH = 10.0) was observed, and after 7 days the hydrodynamic diameter was undetectable by the particle analyzer. The image for such a phenomenon is displayed in Figure S2. Transmission electron microscopy (TEM) confirmed the sizes of nanoparticles under different pH for 7 days, which were consistent with sizes obtained from the nanoparticle analyzer. In Figure 3A, the nanoparticle at day 0 showed a spherical shape with a diameter of around 151 nm in DI water. In the acidic environment (pH = 4.0, Figure 3B), the shape of the nanoparticle was deformed to exhibit a loosely structured, irregular polygonal spherical shape (~207 nm). In the neutral environment (pH = 7.4, Figure 3B), the nanoparticle showed good stability to maintain the spherical shape and size (~153 nm). In addition, the nanoparticles in the alkaline buffer (pH = 10.0) had significant aggregation and failed to retain their shape, as shown in Figure 3D. After optimization, the nanoparticles of APC2 were selected for subsequent drug-loading experiments and referred as APC.

**Figure 4:**
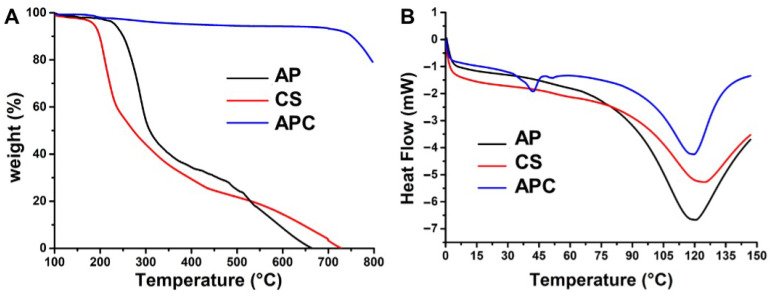
The thermal properties of AP, CS, and APC by (**A**) thermogravimetric analysis and (**B**) differential scanning calorimetry.

The thermal properties of AP, CS, and APC nanoparticles were assessed by thermogravimetric analysis (TGA) and differential scanning calorimetry (DSC). The TGA curves are shown in Figure 4A. The curve of AP revealed that the thermal transition of AP occurred in two temperature ranges, i.e., 255–400 °C and 500–655 °C, which was similar to the thermal transition feature of CS (250–425 °C and 550–725 °C). By contrast, the APC nanoparticle remained thermally stable until the temperature reached 750 °C. The DSC curves of AP, CS, and APC nanoparticles are shown in Figure 4B. The curve of AP exhibited a relatively obvious endothermic broad band at ~120 °C, whereas the curve of CS exhibited an endothermic broad band at ~125 °C. The endothermic value of APC was the smallest among the three groups. In addition to the endothermic absorption band at ~120 °C, an additional small heat absorption peak was observed at 45 °C.

### 2.2. Antibacterial and Antiviral Activities

**Figure 5:**
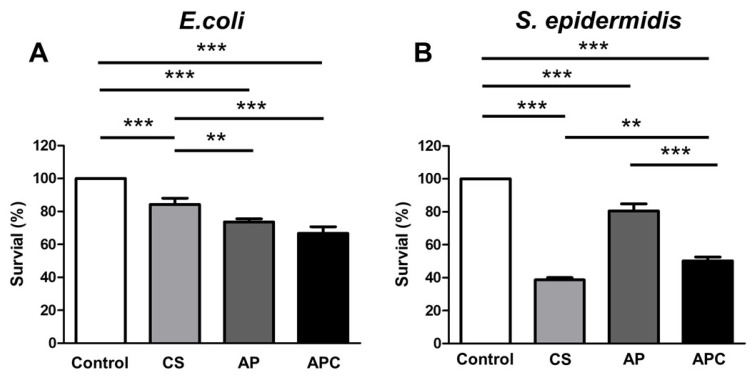
The antibacterial profiles for CS, AP, and APC against (**A**) Gram-negative bacteria (*E. coli*) and (**B**) Gram-positive bacteria (*S. epidermidis*). The number of bacteria was normalized to the absorbance value of the control group (untreated mock bacteria) and expressed as the percentage of survival (%). The ingredients CS (0.86 μg/mL) and AP (1.14 μg/mL) were individually tested for comparison. APC was tested in the form of nanoparticles (2 μg/mL). ** *p* ≤ 0.01 and *** *p* ≤ 0.001 between the indicated groups.

The antibacterial activities of AP, CS, and APC nanoparticles against Gram-negative bacteria (*Escherichia coli*, *E. coli*) and Gram-positive bacteria (*Staphylococcus epidermidis*, *S. epidermidis*) were evaluated. Results from the controlled group, CS, AP, and APC nanoparticles are shown in Figure 5. The data in Figure 5 revealed that CS killed ~17% of *E. coli* and ~60% of *S. epidermidis*, respectively, while AP killed nearly 20% of both types of bacteria without obvious specificity. Compared to the raw materials, APC nanoparticles possessed a better antibacterial activity against *E. coli*, killing ~30%. Although the ability of APC to reduce *S. epidermidis* was slightly inferior to CS, APC nanoparticles still retained a considerable antibacterial effect (killed ~45%).

**Figure 6:**
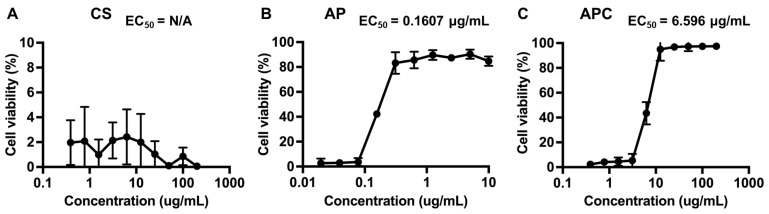
Dose-dependent antiviral activities of CS, AP, and APC. Different concentrations of (**A**) CS, (**B**) AP, and (**C**) APC nanoparticles were added after infection of HSV-2 to Vero cells at 37 °C for evaluation of the antiviral capability and effective concentration. The inhibition efficiency of each group was evaluated by the neutralization test and the living residual Vero cells were expressed as the cells viability rate.

The antiviral evaluations of CS, AP, and APC nanoparticles were performed to examine whether the above material had the ability to inhibit the cytopathic effect of Vero cells induced by Herpes Simplex Virus-2 (HSV-2). The viral titers were assessed using the plaque assay [27]. In Figure 6A, CS was proved to possess no viral inhibitory effect. Both AP and APC nanoparticles showed effective inhibition of the virus and protect cell viability in Figure 6B,C. The survival rate of Vero cells treated with CS was below 2%. This low cell viability obtained from the CS group resulted in the inability to obtain valid half-maximal effective concentration (EC_50_) values. The EC_50_ of the AP treatment cell group is 0.1607 μg/mL, which means that pure AP had the activity to effectively inhibit HSV-2 at a very low content. The EC_50_ of the APC nanoparticles group is 6.596 μg/mL, which was slightly higher than that of the pure AP group, but was also at a relatively low concentration. These results demonstrated that APC remained to have good antiviral performance.

### 2.3. Drug Release and Kinetics of Drug-Loaded Nanoparticles

The drug release profiles for APC nanoparticles loaded with two different model drugs were examined at 37 °C. The fast green-loaded complex nanoparticles (APC-F) were immersed in the phosphate-buffered saline (PBS) buffer, and the curcumin-loaded complex nanoparticles (APC-C) were immersed in PBST (PBS buffer with 5% Tween 20) buffer. The PBST buffer was intended to increase the solubility of APC-C in the environment. Meanwhile, the drug release profile for APC nanoparticles loaded with highly expressible bacteriorhodopsin (HEBR), i.e., APC-H complex nanoparticles, was examined at 4 °C in 2-(N-morpholino)ethanesulfonic acid (MES) buffer. The drug release conditions of APC-H at low temperature were used to preserve the biological activity and prevent protein deterioration of HEBR. Figure S3 is the calibration curve used for the amount of drug released from the drug-loaded APC.

The morphology of three drug-loaded APC nanoparticles re-suspended in DI water at day 0 was observed by TEM, and the images are shown in Figure S4. APC-F exhibited a hollow spherical shape with a diameter of ~189 nm (Figure S4A), which was slightly larger than that of the blank APC nanoparticles. APC-C displayed a solid spherical shape with a diameter of ~65 nm (Figure S4B), which was smaller than that of the blank APC nanoparticles. HEBR is a protein drug with a three-dimensional columnar structure. Based on Figure S4C, the shape of HBER-loaded APC nanoparticles (APC-H) is irregular and the average particle size was ~1420 nm, which was nine times larger than that of the original APC nanoparticle (~151 nm).

The drug release of APC-F and APC-C under different pH values is shown in Figure S5. In Figure S5A, the release profiles of APC-F under the three different pH values were generally similar in the first 12 h. The drug release under pH 7.4 was the fastest in the following 24 h. After 48 h, the release efficiency of APC-F in the acidic environment exceeded that of APC-F in the neutral environment. After 7 days, the release efficiency of APC-F in the acidic environment reached 27%, which was 5% more than that in the neutral environment and 10% more than that in the alkaline environment. In Figure S5B, the release profiles of APC-C under different pH values were similar within the first 24 h. In the following 24 h, APC-C nanoparticles in the pH 10.0 buffer showed a faster drug release rate than those in the other two conditions. After 48 h, the amount of curcumin released from APC-C into the acidic environment was greater than that in the alkaline environment.

**Figure 7:**
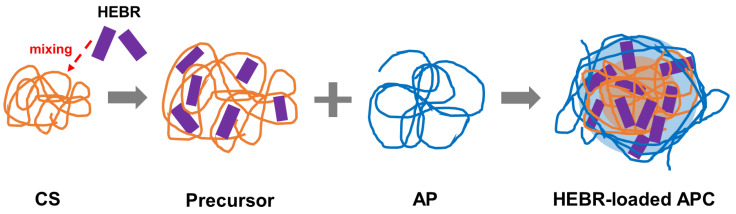
The possible formation process of the HEBR-loaded APC nanoparticles (APC-H complex nanoparticles).

**Figure 8:**
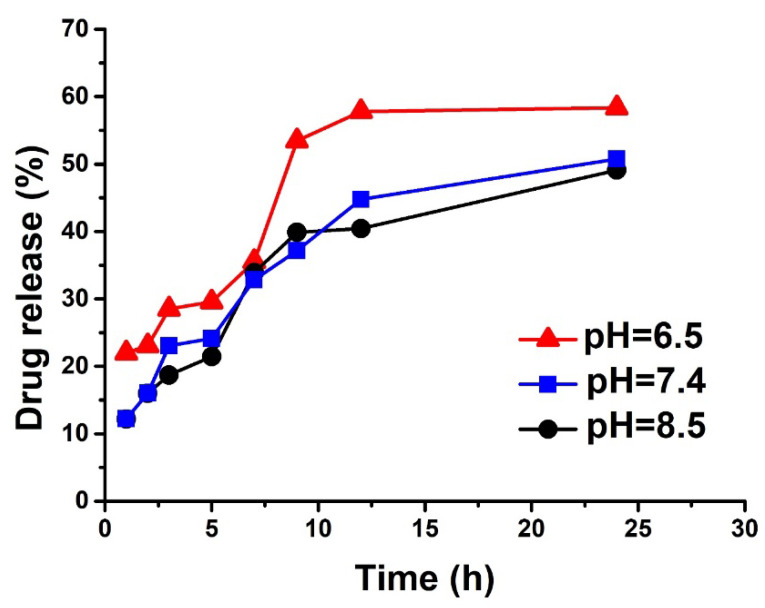
The release profiles of HEBR from the APC-H complex nanoparticles under different pH values for 24 h (in MES buffer).

The possible mechanisms for APC nanoparticles to encapsulate HEBR are illustrated in Figure 7. The release profiles of APC-H at different pH values are presented in Figure 8. Within 24 h, the release efficiency of APC-H in pH 6.5 MES buffer was always higher than that in pH 7.4 and pH 8.5 MES buffers. The releases from APC-H in pH 7.4 and pH 8.5 buffers showed a similar trend. The final release efficiency of APC-H in the acidic environment (~60%) was nearly 10% higher than that in the neutral or alkaline environment.

The fitting data of various kinetic models for all types of drug-loaded APC complex nanoparticles under different environments are summarized in Table 3. R^2^ represents the value of the correlation coefficient. The drug release from drug-loaded nanoparticles was fit with the best correlation using the Korsmeyer–Peppas model, where the correlation coefficients were greater than 0.9 for all groups. The release exponents, i.e., *n*~0.45, were obtained from the Korsmeyer–Peppas model for nanoparticles corresponding to Fickian diffusion.

### 2.4. Functions of HEBR-Loaded Nanoparticles on Cell Behavior

**Figure 9:**
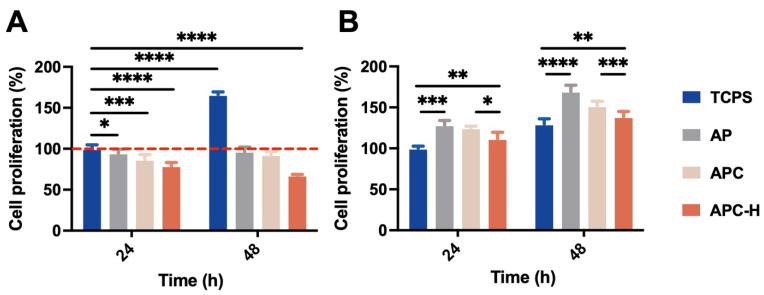
The proliferation of (**A**) A549 lung cancer cells and (**B**) neural stem cells for the TCPS (control group), AP, APC (composite nanoparticles), and APC-H (drug-loaded complex nanoparticles) at 24 and 48 h measured by CCK-8 assay. The cell proliferation was normalized to the absorbance value of the TCPS group at 24 h and expressed as the percentage of cell proliferation (%). * *p* ≤ 0.05, ** *p* ≤ 0.01, *** *p* ≤ 0.001, and **** *p* ≤ 0.0001 between the indicated groups.

According to previous bioactivity data of HEBR on inhibiting certain cell proliferation [23], human epithelial lung cancer cells (A549) and mouse neural stem cells (NSCs) were selected as the target cells for drug release treatments. In Figure 9A, APC-H expressed the best inhibitory effect (with ~40% reduction) on the viability of A549 cells. APC particles alone also exerted a certain extent of inhibition on the growth of A549 cells. Meanwhile, the presence of AP, APC, and APC-H in the cell culture medium showed a promoting effect on NSCs proliferation, as presented in Figure 9B. Among them, APC-H had the smallest promoting effect (Figure 9B), which may be ascribed to the bioactivity of HEBR to inhibit the proliferation of NSCs to some extent [28].

## 3. Discussion

CS is insoluble in neutral water and has to be properly modified or combined with other materials for improving the hydrophilicity and overcoming the limitation caused by the insolubility [29,30]. The preparation process of nanoparticles in the present work is through the electrostatic interaction between the charged groups of two materials, i.e., the negatively charged sulfate groups and carboxylic acid groups in AP together with the positively charged amine groups in CS. Since electrostatic forces are not chemical bonds and would not alter the chemical structure of the material, the original properties of each component can be well retained. AP is a hydrophilic and negatively charged material, and according to the measured zeta potential of the APC nanoparticles (−20.31 mV), the synthesized APC nanoparticles are presumed to be dominated by AP molecules. In addition, the relatively hydrophobic CS molecules were mostly surrounded externally by AP molecules to allow a stable dispersion in the medium. In the optimization process, excessive CS may induce the instability of the APC nanoparticles. When the ratio of CS to AP reached 1.5:1, obvious precipitation was observed in the system. This may be attributed to the hydrophobicity of CS that aggregated the CS chains beyond a certain concentration and resulted in poor suspending ability of composite particles even in the presence of the hydrophilic AP. Similar observations have also been reported in other composite particle systems, where the particle size became larger at the micrometer level with a higher CS ratio and eventually reached the point of aggregation and precipitation [21].

Changes in size for the APC nanoparticles under different pH values were observed. In the physiologically neutral environment (pH = 7.4), the size of the APC nanoparticles was stable, which was surprising. According to a previous report, CS-based nanoparticles composited with SP were unable to retain the structure and could disintegrate when the pH value was above 6.5 [31]. Literature indicated that CS would be deprotonated at a pH value greater than 6.5 and gradually lose its positive charge from the amine groups [32,33]. The decrease in positive charge in CS can affect its binding to other materials due to the reduced electrostatic force [34]. By contrast, the APC nanoparticles in the current work show stable size and morphology at pH above 6.5, which may be associated with the preparation method and the properties of AP. In the present study, CS was ionized in a pH~6.0 surrounding in advance and then combined with swollen AP in a suitable environment. Therefore, the structure of the APC was stabilized by the electric interaction between AP and CS. Except for extreme alkaline environments, the structural changes that are mainly based on CS exfoliations may occur. [35]. In addition, AP possesses good solubility in water. Although CS may undergo deprotonation above pH ~6.5, the structure of the APC nanoparticles is supported by two polymers to maintain the size and structure under a neutral environment. Meanwhile, the main dimensional change in the acidic environment can be attributed to the swelling of CS in the acidic environment [36], while that in the alkaline environment may be due to the structural collapse of CS after deprotonation that leads further to aggregation.

The antibacterial properties of the APC nanoparticles against Gram-positive bacteria (*S. epidermidis*) and Gram-negative bacteria (*E. coli*) were confirmed. CS is generally regarded to have good antibacterial activity against Gram-positive bacteria. The positive charge of CS accounted for its effective inhibition on Gram-positive bacteria [37,38]. In the present work, AP showed growth inhibition on both types of bacteria at similar levels without apparent selectivity. The antibacterial mechanism of AP might be due to the presence of negatively charged groups on AP. These negatively charged groups with polarity may readily chelate or interact with some important cations in bacterial metabolism to disturb the stable hydrogen bonds in the bacterial environment [39,40]. Moreover, the APC nanoparticles exhibited considerable antibacterial properties on both *E. coli* and *S. epidermidis.* The synergistic effect of the APC from CS and AP on *E. coli* and *S. epidermidis* may be attributed to the rupture of the bacterial cell membrane or prevention of the metabolic cycle of the bacteria [41]. In addition, the inhibitory effect of the APC nanoparticles on *S. epidermidis* was slightly worse than that of CS, which may be related to the core-shell-like structure of the APC nanoparticles. Because CS was possibly more concentrated inside the nanoparticles, it could not engage in the antibacterial process. These antibacterial results also verified our hypothesis on the APC nanoparticle structure.

The antiviral evaluation was conducted to see whether CS, AP, and the APC nanoparticles had an effective inhibitory effect on Vero cell pathology induced by HSV-2. Results showed that CS by itself had no effective viral inhibitory effect. On the other hand, AP had an obvious antiviral property because of its abundant negative charge, especially from sulfate groups [42], which can interact with viral particles, inhibit adsorption, hinder its binding to cell receptors, and prevent virus from invading host cells, thus affecting the life cycle of virus [43,44]. In addition, AP can enhance the immune response of the host and induce immune factors to accelerate virus clearance [45,46]. The antiviral effect of the APC nanoparticles was only slightly inferior to that of AP. By comparing the EC_50_ (6.596 μg/mL) of the APC nanoparticles with other materials, the APC nanoparticles demonstrated rather competitive antiviral properties [47,48]. The combination of AP and CS may occupy the sulfate groups of AP and indirectly affect the viral inhibition of the APC nanoparticles. The concentration of sulfate groups on AP was connected to the viral inhibition [49]. In the speculated structure of the nanoparticles, AP is at the periphery and has a greater opportunity to combine and react with virus. In addition, the zeta potential of the APC particles is negative, which implies that some negatively charged groups of AP are not neutralized and are exposed to the external side. Because of the structure, the APC nanoparticles can properly exercise its antiviral function.

The APC nanoparticles, as a multifunctional composite, not only offers beneficial function alone but also has the potential for other applications, such as drug carrier [50,51]. In traditional therapy, medications are commonly used to alleviate the condition in the body. The disease cycle is often associated with complex symptoms and immune deficiencies, as well as complications and side effects after treatment, such as bacterial or viral infections [52,53,54,55]. These symptoms and situations need to be treated at the same time, not with a single-functional drug [56]. In this case, APC nanoparticles with antibacterial and antiviral capabilities can reduce the use of multiple drugs to address the problems of overdose and conflicting drug effects [57,58]. Utilization of the APC nanoparticles may offer a wider range of treatment options within the disease cycle [59]. In this study, three drugs were employed as model drugs representing three different categories of drugs, i.e., fast green for the hydrophilic drug, curcumin for the hydrophobic drug, and HEBR for the macromolecular protein drug, to evaluate the feasibility of encapsulating different types of drugs in the APC nanoparticles. The drug-loading system of the APC nanoparticles possesses a combination of positive and negative charges, as well as hydrophilic and hydrophobic properties of the materials. For example, fast green and HEBR, as negatively charged drugs, interacted with positively charged CS during preparation. The CS–drug complex serving as the hydrophobic core was subsequently covered with the hydrophilic AP chain to further self-assemble into nanoparticles.

The pH-sensitive release behavior of drug-loaded APC nanoparticles was assessed. The drug-loaded APC nanoparticles, regardless of the drug type, showed the best release efficiency under the acidic condition. The morphological and structural changes of non-drug-loaded APC particles after 7 days in the acidic environment infer that AP exhibits a stronger negative charge in the acidic environment and the chain of CS is more expanded to become a looser hydrophobic CS–drug core compared to that in the neutral environment. The loosely structured drug-loaded APC nanoparticles would accelerate the drug release, while the acidic AP would compete with the drug for binding sites on the expanded CS chain to increase the drug release [60]. Similarly, the conformational changes of CS result in sufficient responsiveness of the APC nanoparticles [61]. The lower drug release in a neutral environment may be attributed to the high stability of the APC nanoparticles, which could hinder the release of drug through structural changes. Under alkaline conditions, the CS–drug core in the complex nanoparticles shrinks due to mutual repulsion between the surrounding and the hydrophobic core. Moreover, AP originally robbed some binding sites of drug and CS to promote the drug release. In an alkaline environment, the physical properties of AP make the drug–APC complex nanoparticles susceptible to swelling, which is not conducive to drug diffusion.

The kinetic model was investigated to reveal the release mechanism of drugs from the APC nanoparticles, as summarized in Table 3. The drug release in this study followed several mathematical models, including the first-order kinetics model, Hixson–Crowell model, Higuchi model, and Korsmeyer–Peppas model [62,63,64,65]. After considering the correlation values, the release curves were best fit by the Korsmeyer–Peppas model with a correlation coefficient of ~0.95. Furthermore, Fickian diffusion was confirmed to be the controlling factor of drug release in the APC nanoparticles system via calculating the exponent *n*, which indicated that diffusion is the major mechanism for drug release in the APC system [66]. The suboptimal correlation (R^2^~0.9) of the Higuchi model also supports the argument that the mechanism of drug release in the current system is indeed the Fickian diffusion [67]. The initial fast release might be a result of the dispersed drug molecules near the APC nanoparticle surface that diffused easily in the beginning. After the burst release period, the rate of release decreased as the dominate release mechanism might have changed to polymer erosion [68]. However, the Hixson–Crowell model showed different fitting results for small and large molecule drugs. For small molecule drug, the R^2^ value was found to be below 0.8, which suggests that the surface area of the particles does not change much over time. On the other hand, in protein drug, i.e., the macromolecular drug, the correlation value was above 0.9. One possible reason for this difference is the size difference of the encapsulated drug. There is an obvious size gap between HEBR and the other drug molecules, which explained the distinct change in the surface area of HEBR-loaded nanoparticles.

In this study, the cytotoxicity of AP, APC, and APC-H was evaluated via both A549 cells and NSCs. Considering the size of APC-H nanoparticles (~1420 nm), the cells could not internalize the particles directly. The HEBR may be released from APC-H nanoparticles into the culture medium and then could work on the cells. HEBR-free drug was reported to inhibit the viability of A549 cells to ~60% of the original level after 24-h treatment in previous literature [26]. In the current work, A549 cells were also inhibited after the treatment of APC-H with cell viability reduced to ~75% after 24 h. The effect of APC-H was slightly lower than that of HEBR-free drug. The cell culture conditions were different from in vitro drug release conditions, which might affect the practical drug release from APC-H. However, APC-H still exhibited a considerable inhibition effect on A549 cells (reduced to 60%) after 48 h, which is comparable with the previous findings regarding the strong inhibitory effect of HEBR as a free drug [26]. This result suggests that using the APC nanoparticles as a delivery system for HEBR, which is a bioactive drug, may not adversely affect the therapeutic outcome. Using electrostatics as the drug-loading mechanism may be responsible for the capability of the drug-loaded APC nanoparticles in maintaining the drug activity, i.e., there is no chemical bond to alter the chemical structure of the drug. [69]. Meanwhile, results of NSCs revealed that cell proliferation in all experimental groups was significantly increased by the drug-loaded APC after 48-h treatment. This finding differs from previous reports about the growth inhibition of HEBR on NSCs [70]. AP, a SP, is similar to the extracellular matrix of the human body [71]. AP can be a source of nutrition for cells to promote cell growth by contributing to the cell cycle [72]. Presumably, the presence of AP in APC nanoparticles may reduce the inhibitory effect of HEBR on NSCs growth or, in turn, even enhance the proliferation of NSCs [18,73].

In summary, the APC nanocomposite particles were optimized to have stable size and shape in a neutral environment. They exhibited antibacterial properties against both Gram-positive and Gram-negative bacteria, as well as good antiviral properties. The APC nanoparticles also showed great potential as drug carrier with pH-sensitive release behavior. The release kinetics of drug-loaded APC nanoparticles was investigated for various drugs. The use of APC nanoparticles as a drug delivery system may retain the bioactivity of different types of drugs and relieve the growth inhibitory effect of some drugs on favorable cells.

## 4. Materials and Methods

### 4.1. Preparation of Nanoparticles

The nanoparticles composed of AP/CS, abbreviated as APC, were prepared from AP (Mn ≒ 3 × 10^5^ Da, Far East Bio-Tec. Co., Ltd., Taiwan) and CS (Mn ≒ 1.4 × 10^5^ Da, Hopax lnc., Taiwan). Two solutions were prepared first. CS was first dissolved in 1 wt% acetic acid (Sigma-Aldrich, St. Louis, MO, USA) with a concentration of 10 mg/mL. The highly concentrated CS solution was then diluted with PBS to the targeted concentrations (0.5, 0.75, and 1 mg/mL) at a pH value of around 6.0. The AP solution with a concentration of 1.0 mg/mL was dissolved in DI water. The AP aqueous solution was dropped slowly into the CS solution with different concentrations under the mixing speed of 800 rpm. The whole preparation process of APC was conducted at room temperature. After mixing the two solutions, the preliminary mixture was centrifuged at 5000 rpm for 15 min. The precipitate was suspended in DI water and recentrifuged. This process was repeated three times. The final sedimentation was the target APC nanoparticles.

### 4.2. The Structure and Properties of Nanoparticles

The PDI, hydrodynamic diameter, and zeta potential were determined by a nanoparticle analyzer (Delsa Nano, Beckman Counter, Brea, CA, USA) at 25 °C. The morphology and average size of APC nanoparticles were observed with TEM (H-7100, Hitachi Ltd., Tokyo, Japan) and estimated by image analysis. Furthermore, the thermal stability and weight loss of the nanoparticle were verified by TGA (Q50, TA Instruments, New Castle, DE, USA) heated from 25 °C to 800 °C with the heating speed of 10 °C/min. DSC (Q-20, TA Instruments, New Castle, DE, USA) measurements were recorded during heating to 150 °C for AP, CS, and APC nanoparticles.

### 4.3. Antibacterial Evaluation

The antibacterial activities of AP, CS, and APC nanoparticles were tested using the standard microdilution method, which determines the minimum inhibitory concentration (MIC). The test bacteria were *Staphylococcus epidermidis* (*S. epidermidis*; ATCC^®^12228TM) and *Escherichia coli* (*E. coli*; ATCC^®^23815TM), respectively, as Gram-positive bacteria and Gram-negative bacteria. These bacteria were cultured in Luria–Bertani (LB) broth at 37 °C for 1 day and diluted to 1 × 10^6^ CFU/mL with fresh LB broth. CS was first dissolved in 1 wt% acetic acid with a concentration of 10 mg/mL as a stock. The stock was then diluted with LB broth to 0.86 μg/mL. AP was directly dissolved in LB, and APC nanoparticles were directly suspended in LB broth to achieve the desired concentration (1.14 μg/mL for AP and 2 μg/mL for APC nanoparticles, respectively). AP, CS, and APC nanoparticles were placed in a 24-well plate, and 1 mL of diluted bacteria solution was added to each well and incubated at 37 °C for 1 day. The solution was read at 595 nm by a microplate reader (Model 680, Bio-Rad Laboratories Inc., Hercules, CA, USA). The controlled group was the bacteria cultured in the LB broth without any treatment.

### 4.4. Antiviral Assessment

The antiviral evaluation was carried out according to the neutralization test, where the inhibition of the cytopathic effect induced by HSV-2 on Vero cells was measured. Vero cells in 96-well plates with a density of 10,000 cells per well were infected with HSV-2 virus (plaque-forming unit 0.15) and were treated with serial concentrations of CS, AP, or APC nanoparticles in the medium composed of high-glucose Dulbecco’s modified Eagle’s medium (HG-DMEM, Gibco, Grand Island, NY, USA) supplemented with 2 wt% of fetal bovine serum (FBS, Gibco, Grand Island, NY, USA). Cells were first incubated at 37 °C for 96 h. Then, the plates were fixed by adding 0.5 wt% of formaldehyde and were incubated for 4 h at room temperature. Thereafter, the formaldehyde was removed and the wells were stained with 0.1 wt% crystal violet for 10 min at room temperature. Plates were washed and air-dried, and the cellular densities of each well were measured with a microplate reader (BioTek, Winooski, VT, USA) at 570 nm. The concentration of CS, AP, or APC nanoparticles required to reduce virus-induced cytopathic effects by 50% compared to untreated controls was expressed as EC_50_.

### 4.5. Preparation of the Drug-Loaded APC

The AP and CS solutions were sonicated. For loading the model drug, 0.0025 g of fast green (Sigma-Aldrich, St. Louis, MO, USA), curcumin (Sigma-Aldrich, St. Louis, MO, USA), or the photosensitive protein (HEBR) was first added to the CS solution. Mass production of HEBR was established based on the *E. coli* system according to previous literature [70,74]. All HEBR-related experiments were prevented from light. The AP solution was dropped slowly into the CS-drug solution (0.75 mg/mL) and mixed at 800 rpm for drug loading and self-assembly into the nanoparticulate form. The whole preparation process of drug-loaded nanoparticles was conducted at room temperature. The final mixture was centrifuged at 2500 rpm for 15 min. The procedures varied for different categories of drugs, i.e., fast green as the hydrophilic drug, curcumin as the hydrophobic drug, and HEBR as the protein. Considering the dissolvability of the hydrophilic drug and protein, the sediment at the bottom of the centrifuge bottle was APC-F and APC-H. Meanwhile, the hydrophobic curcumin was loaded by a different protocol. The precipitates obtained by centrifugation were resuspended, and the suspension was settled for 1 h to obtain the homogeneous supernatant, i.e., APC-C. The morphology and average size of drug-loaded APC were examined by TEM.

### 4.6. Drug Loading and Encapsulation Efficiencies

The loading and encapsulation efficiencies of the nanoparticles were determined. The analytical procedures slightly varied based on the different drug-loading methods. As for the hydrophilic and protein drugs, the weight of resident drugs in the supernatant liquid from centrifuged solution was measured to calculate the non-loaded fast green or HEBR. Because of the rather complicated loading process of the hydrophobic drug, the weight of non-loaded drugs was divided into two portions, including the resident drug in the supernatant after centrifugation and in the precipitation from the resuspended solution. The non-loaded curcumin in both parts was measured and recorded. The amount of non-loaded drug in the supernatant was measured using a microplate reader (SpectraMax M5, Molecular Devices, San Jose, CA, USA) to obtain the absorbance at the specific wavenumber for each drug, i.e., 425 nm for curcumin, 551 nm for HEBR, and 622 nm for fast green. The concentration of the residual drug in the supernatant was first calculated by taking the measured absorbance into the quantitative curve of each drug, and then the weight of the residual drug was obtained based on the volume of the supernatant and the concentration of the drug. The precipitate from the resuspended solution was dried to determine the weight of a portion of the non-loaded curcumin. The drug loading and encapsulation efficiencies of the nanoparticles were calculated as follows:

(1) $$\mathrm{Loading}\;\mathrm{efficiency}\;=\frac{W_{I}-W_{U}}{the\;weight\;of\;nanoparticle}\times 100\%\;$$

(2) $$\mathrm{Encapsulation}\;\mathrm{efficiency}\;=\frac{W_{I}-W_{U}}{W_{I}}\times 100\%\;$$

where *W_I_* is the initial weight of the drug used to prepare the nanoparticles and *W_U_* is the total weight of the non-loaded drug.

### 4.7. Release Efficiency and Kinetics of Drug-Loaded Nanoparticles

Drug release from the APC-F and APC-C nanoparticles was studied at 37 °C. Because of the thermostability of HEBR, drug release from APC-H was studied at 4 °C. The ambient solution used in these experiments was PBS for APC-F and PBST solution for APC-C, at different pH values of 4.0, 7.4, and 10.0. Considering the activity of protein, the MES buffer was selected as the release environment for APC-H, and the pH variations of MES buffer solutions were 6.5, 7.4, and 8.5. In the case of APC-F and APC-C, 11 mg of the nanoparticles was added to 75 mL of PBS or PBST. As for APC-H, 3 mL of MES buffer solution was added. The weight of the drug released into the ambient solution was achieved at the preset time using the same calculation method through the absorbance measured by the microplate reader mentioned in Section 4.6. The drug release ratio from the nanoparticles was calculated as follows:

(3) $$\mathrm{Drug}\;\mathrm{release}=\frac{W_{a}}{W_{0}}\times 100\%$$

where *W_a_* is the weight of the drug released from the nanoparticles into the ambient solution at preset time points and *W*_0_ is the total weight of the unloaded drug.

### 4.8. Cell Viability and Proliferation

The Cell Counting Kit-8 (CCK-8, Sigma-Aldrich, St. Louis, MO, USA) assay was used to estimate the cell viability and proliferation of nanoparticles. NSCs, which were derived from the adult mouse brain [75], were prepared in culture medium to get the cell suspension and count. The culture medium was HG-DMEM and Ham’s F-12 (Gibco, Grand Island, NY, USA) supplemented with 10% of FBS, 400 mg/mL G418 (Invitrogen, Waltham, MA, USA), and 1% penicillin–streptomycin–amphotericin (Gibco, Grand Island, NY, USA). Meanwhile, A549 cells were cultured in the Roswell Park Memorial Institute medium (RPMI 1640, Gibco, Grand Island, NY, USA). The culture media contained 1% penicillin–streptomycin (Gibco, Grand Island, NY, USA) and 10% FBS.

The NSCs or cancer cells were seeded in 24-well plates with a density of 8 × 10^3^ cells per well. Then, AP (80 μg/mL), APC nanoparticles (140 μg/mL), or APC-H (180 μg/mL, of which HEBR content accounts for 40 μg/mL) were added. After 24 and 48 h, 300 μL of CCK-8 solution was added to each well. The supernatant of each well was measured at 450 nm by the microplate reader (SpectraMax M5, Molecular Devices, San Jose, CA, USA). Cells cultured on tissue culture polystyrene plate (TCPS) were employed as the control group.

### 4.9. Statistical Analyses

Data from experiments were expressed as mean ± standard deviation. The statistical analysis of data was performed by one-way analysis of variance (ANOVA). A *p*-value of less than 0.05 was considered statistically significant.

## 5. Conclusions

Biocompatible nanoparticles, i.e., APC, composed of AP and CS were successfully developed and optimized to obtain the stable morphology and size of about 160 nm. The pH sensitivity of the APC nanoparticles and the changes in both shape and size were well confirmed under surroundings of different pH values. APC nanoparticles exhibited non-specific antibacterial properties against Gram-positive bacteria (killed ~45%) and Gram-negative bacteria (killed ~30%). In addition, the antiviral function of APC nanoparticles was maintained with the half-maximal effective concentration of 6.596 μg/mL. APC nanoparticles featured a combination of the properties of each component. In vitro release studies and mathematical modeling of the release kinetics for the drug–APC complex demonstrated the increased release rate of drug in acidic conditions (pH = 4.0) due to swelling and diffusion. At the same time, three different kinds of drugs were used to investigate the potential of APC composite nanoparticles as drug carriers. Furthermore, in vitro cell studies of drug-loaded APC nanoparticles (APC-H complex), taking HEBR protein drug as an example, were conducted using A549 cells and NSCs. The APC-H nanoparticles effectively prevented the proliferation of A549 cells with the cell viability reduced to ~60%, a contribution mainly from HEBR. APC-H also showed a slight promotive effect on NSCs proliferation, suggesting that APC could alleviate the inhibitory effect of the drug on favorable cells. These findings support the promising application of multifunctional APC nanoparticles as potential drug carriers in medication treatment and delivery.

## Figures and Tables

**Table 1:** Compositions for various AP/CS (abbreviated as APC) nanoparticles prepared with various mass ratios of AP and CS.

Abbreviated Names	AP /mg mL^−1^	CS /mg mL^−1^
APC1	1.0	0.5
APC2	1.0	0.75
APC3	1.0	1.0

**Table 2:** The zeta potentials and polydispersity index (PDI) of APC nanoparticles prepared with various mass ratios of AP and CS.

Samples	Zeta Potential	PDI
APC1	−17.3 ± 1.2 mV	0.278
APC2	−20.31 ± 1.6 mV	0.256
APC3	−16.6 ± 0.9 mV	0.271

**Table 3:** Release kinetics for different drugs from the drug–APC complex nanoparticles (APC-F, APC-C, and APC-H) under different pH values, with R^2^ as the correlation coefficient value for each kinetic model.

Drug	pH Value	R^2^			
		1st-Order	Hixson–Crowell’s	Higuchi’s	Korsmeyer–Peppas’s
Fast green	4.0	0.8689	0.7976	0.9419	0.9619
	7.4	0.6127	0.6445	0.8710	0.9578
	10.0	0.6780	0.7437	0.8922	0.9474
Curcumin	4.0	0.9215	0.9000	0.9354	0.9539
	7.4	0.8801	0.8610	0.9448	0.9922
	10.0	0.7690	0.7163	0.9073	0.9681
HEBR	6.5	0.8511	0.8769	0.9082	0.9278
	7.4	0.9703	0.9042	0.9723	0.9765
	8.5	0.9329	0.9082	0.9389	0.9470

## Data Availability

Not applicable.

## Supplementary Materials

The following supporting information can be downloaded at: https://www.mdpi.com/article/10.3390/molecules28052105/s1. Figure S1: Precipitation was observed for APC at the AP:CS weight ratio of 1:1.5. Figure S2: Precipitation occurred for APC2 in alkaline PBS solution (pH = 10) after 7 days. Figure S3: The calibration curves of fast green, curcumin, and HEBR. Figure S4: TEM images of initial APC-F, APC-C, and APC-H nanoparticles. Figure S5. Release profiles of different drug-loaded APC particles.

## Sample Availability

Samples of the compounds are not available from the authors.
