# Supplementary material for: Antiviral and Antibacterial Sulfated Polysaccharide–Chitosan Nanocomposite Particles as a Drug Carrier
Source: Molecules. 2023 Feb 23;28(5):2105. doi: 10.3390/molecules28052105 (PMC10003885; doi:10.3390/molecules28052105)
Supplement: Supplementary file 1 [file molecules-28-02105-s001.zip › molecules-2229506-supplementary.pdf]

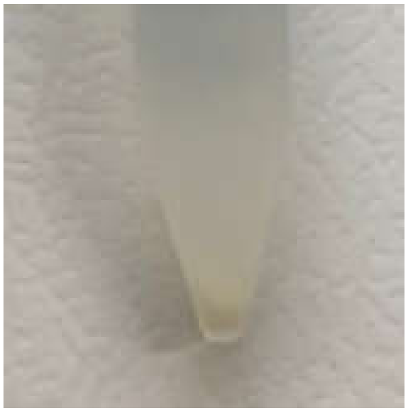

Figure S1. Precipitation was observed for APC at the AP: CS weight ratio of 1:1.5.

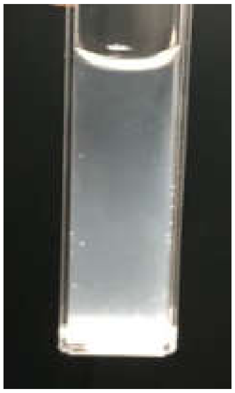

Figure S2. Precipitation occurred for APC2 in alkaline PBS solution (pH = 10) after 7 days.

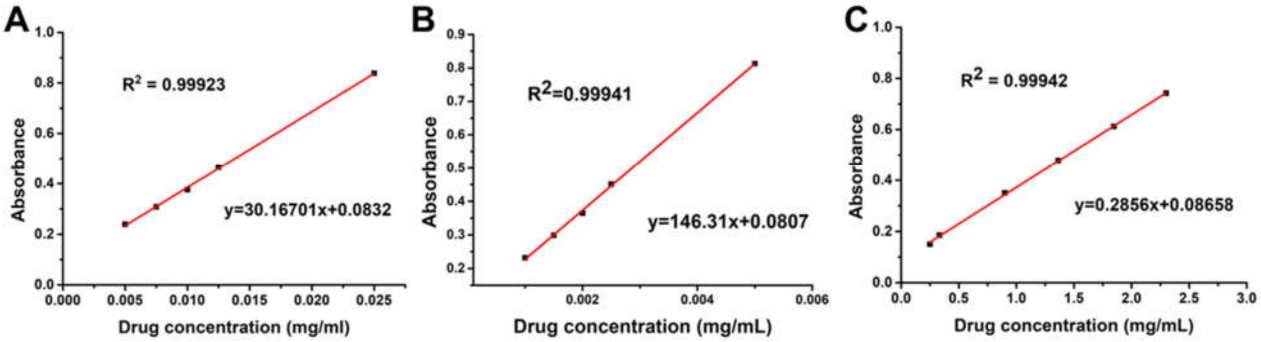

Figure S3. The calibration curves of fast green, curcumin, and HEBR.

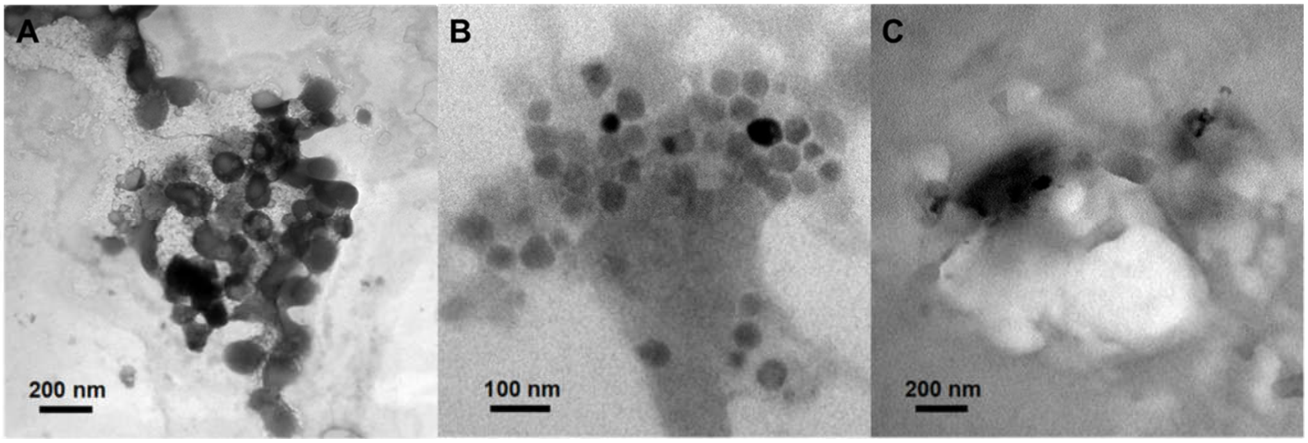

Figure S4. TEM images of initial drug-loaded APC nanoparticles (APC-F, APC-C, and APC-H). All drug-APC complex nanoparticles were prepared in DI water and observed at day 0.

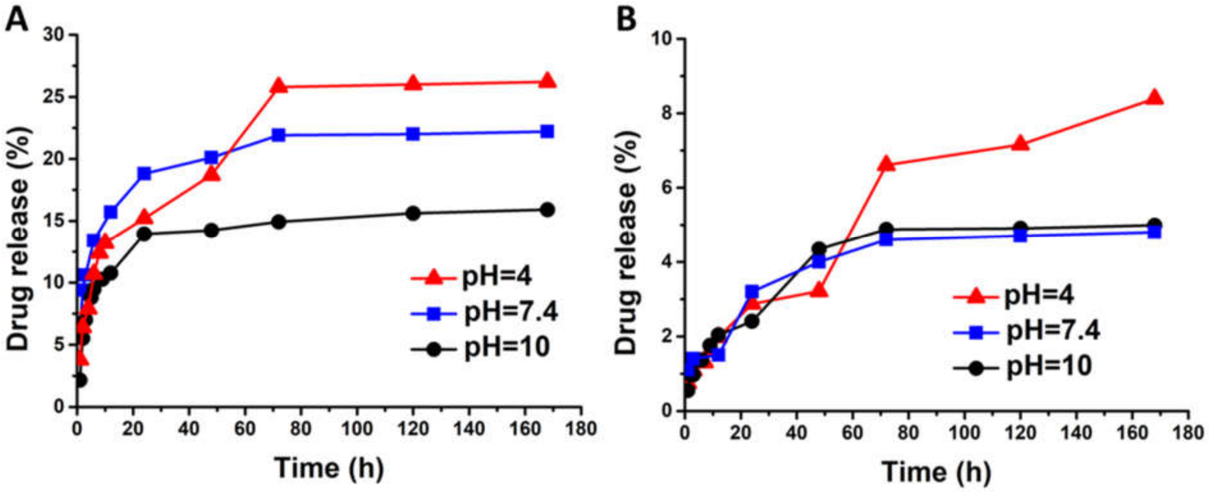

Figure S5. Release profiles of different drug-loaded APC nanoparticles. The release curves of (**A**) fast green and (**B**) curcumin from APC-F and APC-C nanoparticles respectively under different pH values.
